# Wireless Measurement of Sympathetic Arousal During *in vivo* Occupational Therapy Sessions

**DOI:** 10.3389/fnint.2020.539875

**Published:** 2020-10-29

**Authors:** Elliot Hedman, Sarah A. Schoen, Lucy J. Miller, Rosalind Picard

**Affiliations:** ^1^mPath, Boulder, CO, United States; ^2^STAR Institute for Sensory Processing Disorder, Centennial, CO, United States; ^3^Rocky Mountain University of Health Professions, Provo, UT, United States; ^4^Massachusetts Institute of Technology, Cambridge, MA, United States

**Keywords:** sensory processing disorder, sensory modulation disorder, arousal, technology, sympathetic activity, electrodermal activity

## Abstract

**Purpose:**

One goal of occupational therapists working with children who have sensory processing challenges is the regulation of arousal. Regulation strategies have not been evaluated using an empirical measure of physiological arousal.

**Objective:**

To establish the feasibility of using an objective physiologic measure of sympathetic arousal in therapeutic settings and explore the relation between therapeutic activities and sympathetic arousal. To evaluate changes in electrodermal activity (EDA) during occupational therapy sessions.

**Methods:**

Twenty-two children identified with sensory modulation dysfunction (SMD) wore a wireless EDA sensor during 50 min occupational therapy sessions (*n* = 77 sessions).

**Results:**

All children were able to wear the sensor on the lower calf without being distracted by the device. The five insights below are based on a comparison of EDA recordings in relation to therapists’ reflections describing how sympathetic arousal might correspond to therapeutic activities.

**Conclusion:**

Objective physiological assessment of a child’s sympathetic arousal during therapy is possible using a wireless EDA measurement system. Changes in EDA may correspond directly with therapeutic activities. The article provides a foundation for designing future therapeutic studies that include continuous measures of EDA.

## Introduction

Children with developmental disorders such as autism spectrum disorder (ASD) and attention-deficit/hyperactivity disorder (ADHD) often have challenges in sensory processing, and these sensory issues may occur in the absence of other formal diagnoses (Goldsmith et al., 2006; Carter et al., 2011). People with sensory processing challenges have difficulty detecting, regulating, and/or interpreting sensory information and often have difficulty making appropriate responses to sensory input (Miller et al., 2007). Five percent (Ahn et al., 2004) to 16.5% (Ben-Sasson et al., 2009) of children have been shown to be affected by sensory processing challenges. One of the three primary classifications within the sensory processing taxonomy is sensory modulation dysfunction (SMD), wherein individuals have difficulty regulating their responses to sensory stimuli (Miller et al., 2007).

Being able to take in, organize, and interpret different kinds of sensations is critical to function in daily life. When this process is distorted and sensation is perceived unreliably or inaccurately, then everyday encounters become confusing and overwhelming, often resulting in physiologically based dysregulation (Miller, 2014). A common feature of all SMD subtypes is behavioral manifestations of arousal problems, noted by dysregulated behavior, foundational to maintaining attention and achieving an optimal level of function (Miller et al., 2007). Previous research suggests that atypical responses of children with SMD are associated with abnormal functioning of the sympathetic nervous system (Mangeot et al., 2001) and/or the parasympathetic nervous system (Schaaf et al., 2003). Those children with sensory overresponsivity are exceedingly sensitive, which manifests as responding too quickly, too frequently, and/or for too long a time to specific sensory stimuli (Reynolds and Lane, 2008). These children are often hypervigilant to sensory events and appear hyperaroused particularly when incoming sensory information is unpredictable. Children with sensory underresponsivity seem oblivious to many types of stimuli and have difficulty attending to incoming sensory information. These children often appear hypoaroused. Dysregulation may correlate with atypical autonomic/sympathetic nervous system arousal that can impact an individual’s ability to respond in a flexible and adaptive manner to daily experiences (Gunnar and Quevedo, 2007).

Many children with developmental and behavioral disorders have sensory modulation challenges (e.g., National Research Council, 2001; Prizant et al., 2003; Siegel and Solomon, 2003; Greenspan and Wieder, 2007a). Because sensory modulation reflects the ability to regulate or adjust one’s behavior in response to the demands and expectations of the environment, it affects participation in the occupations of daily life (Chien et al., 2016). Arousal dysregulation as a result of sensory modulation challenges appears related to dysfunction across a wide range of areas including social participation, academic performance, self-care, self-esteem, and self-confidence (Bar-Shalita et al., 2008; Cosbey et al., 2010; Cohn et al., 2014; Ismael et al., 2015) across the lifespan.

The relation between sensory modulation and arousal is important knowledge to investigate as it supports providing regulation strategies to children with sensory processing challenges (Reebye and Stalker, 2007). A primary focus of therapy is regulating arousal through creating a sense of safety in the environment and in the therapeutic relationship while addressing specific presenting concerns (Miller et al., 2018; Schoen et al., 2018). Based on the work of Hebb (1949, 1955), an important first step is achieving an optimal level of arousal, which is linked to achieving a maximal level of performance, as overarousal or underarousal is postulated to have a direct negative relationship with performance. An optimal level of arousal maximizes the opportunity for a child to observe and process information needed for cognitive and other executive functions (Greenspan and Wieder, 2008), as well as emotional processing and play (Gunnar and Quevedo, 2007).

One way arousal is evaluated is by conducting a Likert scale survey or interview during therapy (e.g., see Thayer, 1967). When a child becomes overwhelmed, their behavior can change: a child may hide under a table or be unable to speak, suggesting he/she may have a higher arousal. But determining when and how much a child’s arousal changes within each intervention session is difficult. Children, in particular, have challenges expressing their emotions and verbalizing response to treatment (Ammentorp et al., 2006). Children diagnosed with ASD, who frequently have sensory modulation challenges, also have difficulty identifying and describing their emotional state (Hill et al., 2004; Gaigg et al., 2018). In a more recent study, individuals with and without ASD who had greater difficulty identifying and describing their feelings had lower peripheral skin conductance responses, as well as a lower correlation between their subjectively reported and objectively measured level of arousal (Gaigg et al., 2018).

Another method to evaluate arousal is presumed by observations of a client’s behavior. However, visible behavioral cues do not always match the child’s internal arousal (Li et al., 2015; Zantinge et al., 2018): a child may be sitting still and looking calm while his or her arousal is high or is increasing dramatically. The difference between outward behavior and internal arousal is a result of many factors, including individual differences and contextual factors. These factors can make inferring physiological arousal states via behavioral observation imprecise.

A more direct/objective way to assess arousal is recording biological signals. As one becomes aroused, the sympathetic nervous system activates. Many measurable biological signals change with sympathetic activation including electrodermal activity (EDA), blood pressure, heart rate, and pupil dilation (Critchley et al., 2013). EDA is a measure that takes advantage of sweat excreted by the eccrine glands, innervated solely by the sympathetic nervous system (Dawson et al., 2000). Measures of EDA are frequently used as an indicator of changes in sympathetic arousal. Laboratory studies with this same population have shown atypical levels of arousal as measured by EDA and vagal tone in children with SMD. Previous studies have shown that children with SMD may have atypical levels of EDA in response to sensory stimuli (Mangeot et al., 2001; Reynolds and Lane, 2008; Schoen et al., 2008a), as well as atypical levels of vagal tone (Schaaf studies). Research provides increasing confidence of the reliability of this measure in naturalistic setting and suggests that these children may have measurable changes in EDA during sensory-based occupational therapy.

Mitigating the limitations of laboratory-based measures can be achieved by sampling *in vivo* (in natural settings) (e.g., Wilhelm and Roth, 2001; Teller, 2004). Since these data are acquired in a more ecologically valid context the results likely are more informative than may be discovered in an artificial laboratory setting (Fahrenberg et al., 2007). Similar to the methodology used in this study, ambulatory/wireless devices are increasingly being used in *in situ* studies, e.g., stress of employees (Hernandez et al., 2011), frustration of mothers when learning a game (Hedman, 2011), the likelihood of seizures in children with epilepsy (Poh et al., 2012) and presence of atypical sleep patterns (Sano and Picard, 2011). Wearable devices measuring continuous autonomic and physical activity data have contributed important data for medical studies in neurology (Onorati et al., 2017) even leading to FDA certifications of wearable devices that are now worn 24/7 by thousands of patients with epilepsy (Regalia et al., 2019).

Although several studies have measured the physiological arousal of children with sensory processing issues in a laboratory setting, there are no published studies on the feasibility, applicability, and utility of measuring physiological arousal during typical occupational therapy sessions. Thus, this study had two aims:

1) To determine if EDA could be unobtrusively and accurately measured *in situ* in children with atypical sensory modulation during occupational therapy sessions using a wireless sensor and
2) To explore the relations between therapeutic activities/engagement and changes in EDA during occupational therapy for children with sensory modulation challenges.

This study also proposed to employ a new approach to the investigation of a commonly observed aspect of occupational therapy practice since states of arousal are essential to evaluate while working on higher level functional abilities. Thus, a case study methodology was deemed most appropriate for exploration of the data. This methodology draws on the depth of experience of the clinical practitioner and supports the development of future research questions that would be answered with more rigorous designs (Budgell, 2008). Additionally, this approach allows for the evaluation of intervention effects within a single session, as well as enabling modifications if the intervention is not working as planned (Lane et al., 2017). Like single case designs, case studies offer a way of understanding a phenomena that may not have been previously explored. This study makes no causal claims but rather presents behavioral and physiological findings in the form of “insights.”

## Materials and Methods

### Participants

Twenty-three children completed the study. Children were recruited from the Sensory Therapies and Research (STAR) Institute in Greenwood Village, Colorado. Children were referred for participation in the study by their occupational therapist following a comprehensive evaluation. The children were considered a good candidate if they were identified as having sensory modulation challenges which was confirmed after completion of two or more therapy sessions. Parents of children signed written informed consent and children older than seven signed an assent form. All procedures were previously approved by the Internal Review Boards of the Massachusetts Institute of Technology and Rocky Mountain University of Health Professions.

Children participated in a 2 h comprehensive occupational therapy evaluation at the STAR Institute, which included standardized scales of motor performance, observations in the occupational therapy gym, and standardized parent report questionnaires. Based on this information and global clinical impression, all children were identified by expert occupational therapists as having sensory modulation challenges.

One child withdrew from the study because his therapist felt that videotaping was disruptive to his therapy (not related to wearing the sensors). Demographic information about the remaining 22 children is provided in Table 1.

**TABLE 1:** Demographic characteristics of sample (*n* = 22).

**Characteristics**	**n**	**%**
**Gender**		
Male	14	64
Female	8	36
**Age (y)**		
3–4	3	14
5–6	9	24
7–8	7	32
9	3	14
**Ethnicity**		
Caucasian	20	91
Hispanic	2	9
Parent’s education		
College	22	100
**Comorbidities**		
ADHD symptoms	5	23
Anxiety symptoms	2	9
Miscellaneous*	4	18
**Medications**		
Homeopathic	1	5
Antipsychotic	1	5
Stimulant	2	9
Antihypertensive	1	5

*Four children had one of the following: cognitive delay, disruptive behavior disorder, mood disorder, and immature neurological development.

### Data Collection Device

A newly developed and validated sensor was used to record EDA wirelessly in therapy (Fletcher et al., 2010). This sensor was a beta version of the Empatica E4 wearable wristband device for the real time acquisition of EDA data acquisition in real time launched in 2017^1^. This sensor does not interfere with activity; thus, children and therapists could participate in therapy as usual, while physiological arousal data were collected, without child or therapist being aware of the data collection after they habituated to the device. The sensor used 1.5 mm Ag–AgCl electrodes without gel and had been used in other *in situ* studies: (Poh et al., 2010; Hedman, 2011; Hernandez et al., 2011; Sano and Picard, 2011).

EDA is traditionally measured on the palm, fingers, or soles of the feet (Edelberg, 1967; Venables and Christie, 1980). For this experiment, children wore the sensors inside a snug sweatband on the bottom of the calf, above the moving parts of the ankle, resulting in minimal movement of the sensor even when the children twisted and moved their feet. Research suggests that EDA measured from the bottom of the calf and EDA from the palm are moderately correlated (in adults *r* = 0.496, *n* = 17) (van Dooren and Janssen, 2012). Unpublished data from a pilot study conducted before initiation of this research showed a correlation of *r* = 0.75 between palm and calf recordings (Hedman, 2010). Additionally, data from a more recent study showed a range from *r* = 0.75 to *r* = 0.88 for data collected from the calf compared to palm (Fedor and Picard, 2014).

### Procedure

Children were videotaped and time-stamped EDA was measured continuously throughout the 50 min OT session. Children arrived 15 min prior to their occupational therapy session to place the sensors, allowing time for the children to acclimate to the sensors. One research assistant videotaped the therapy session and the other monitored data collection in real time on a portable computer. Data were collected from the lower calf of each leg, positioned above the moving parts of the ankle joint. However, to analyze data, only one sensor’s data were analyzed.

Children received occupational therapy using the STAR PROCESS, a short term, intensive treatment approach that facilitates developmental changes in children with sensory processing challenges. The manual for this approach appears in several publications (Miller, 2014; Miller et al., 2018). The theoretical foundation for treatment is derived from sensory integration (Ayres, 1972) and DIR/Floortime (Greenspan and Wieder, 2007b). The program is unique for its frequency and intensity of delivery (50 min sessions, offered 3–5 times a week for 6–10 weeks), its inclusion of a significant parent collaboration component, and its focus on arousal regulation as a foundation for engagement and relationships and sensory processing. Twenty percent of the sessions are parent- only meetings. Treatment goals are based on parent priorities and typically focus on social participation, self-regulation, and self-esteem (Cohn et al., 2014). Therapy is individualized to the needs of the child through the process of clinical reasoning based on responses/reactions to therapy experiences and challenges. A wide variety of therapeutic interactions occur depending on child’s needs and context of activity, ranging from sitting relatively still to paint, eat, or plan the session to extensive movement e.g., climbing a rock wall, riding a zip line, playing in a ball pit or jumping on a trampoline. Included in some STAR PROCESS therapy programs is the iLs Voice Pro, part of the Integrated Listening Therapy^®^ intervention. The iLs Voice Pro^TM^ is designed to improve an individual’s ability to process sound efficiently and accurately; thus, it is often used as a social training tool in occupational therapy. During this activity, children wear headphones and hear their own voice when they talk into a microphone. Fidelity to the treatment approach was attained through weekly videotaped review of treatment sessions during individual supervision and team meetings.

Therapists facilitated interpretation of data using a participatory design context (Schuler and Namioka, 1993). As the EDA was recorded live, therapists could view the recordings in real time. Videos of the children in therapy were displayed at the same time as EDA. This helped the therapists re-watch the therapy session and better understand how therapeutic methods and EDA corresponded. Video review is a common procedure used in occupational therapy for the purpose of clinical reasoning during which problems, plans or responses to treatment are processed. Thus there was not intent to assess inter-rater agreement during video review.

#### Data Collection/Variables

The beta version Empatica sensor was used for EDA data acquisition. Data was collected continuously throughout the 50 OT session. Children were videotaped and EDA was time-stamped to align with the video. No other routine data were collected on the participants.

The data collection program (similar to that used by the Empatica E4 device) allowed researchers to conduct in-depth analysis and visualization of all variables. This data collection program was used by Picard (2020) with a sample of children on the autism spectrum. A Supplementary Data File depicts the range and variability of EDA responses in that sample. Variables included number of successful recordings, number of children able to complete the study, percent of missing data, mean EDA signal level, and ability to meaningfully associate EDA levels with observed behavior. Data were used from the more responsive side of the body, e.g., the side with the larger mean average EDA across the sessions. No session was used unless a child’s skin conductance level reached the threshold of 0.5 μS. This threshold was based on long-standing recommendations in the physiology literature (Dawson et al., 2000). Values were checked for threshold, and were filtered for noise; data were then only used for analysis when 80% or more were collected without wireless dropouts. A Dell computer was used to receive the signal from the EDA sensor. The research assistant controlling the computer was is no more than 10 feet away from the child and therapist during the treatment session and required an unobstructed view for transmission of data to occur.

## Results

### Feasibility

EDA was successfully measured wirelessly during therapy for all 22 participants without requiring the therapist to modify any of the activities. Seventy-seven hours of recorded video and corresponding EDA were collected. No child participating in the study asked for the sensors to be removed. The acceptance of the sensors is particularly noteworthy given that many of the children had overresponsivity to touch stimuli. Children treated the sensors like socks putting them on with minimal resistance. The sensors were out of the children’s eyesight so the children did not focus on them or bend down to adjust their sensors. In fact, some children forgot to take the sensors off as they left, and the therapists needed to remind them to remove the sensors. Only one participant had to be withdrawn from the study because his therapist noted that the child behaved differently when the research assistants were running the study in the room (an issue separate from use of the sensors).

Data from 21% of the sessions were unusable due to sensor malfunction or EDA too low to detect change (e.g., EDA data were discarded if it was below the threshold of 1 μS). Technical problems included low batteries in one of the sensors and an obstructed signal from the sensor to the PC. Both sensors only stopped recording simultaneously 2.6% of total recorded hours. Obstruction, which prevented wireless transmission and resulted in missing data, occurred when the child sat with their legs crossed (e.g., covering the sensor), or when the child was engaged in an activity such as playing in the ball pit or hiding under a large crash pillow.

Mean EDA across children was 2.50 *u mhos* with a standard deviation of 3.39 *u mhos*. Children with low amplitude EDA, (e.g., between.92 and 1.67 *u mhos*), also had small amplitude changes making data analysis difficult (Edelberg, 1972).

**FIGURE 1:**
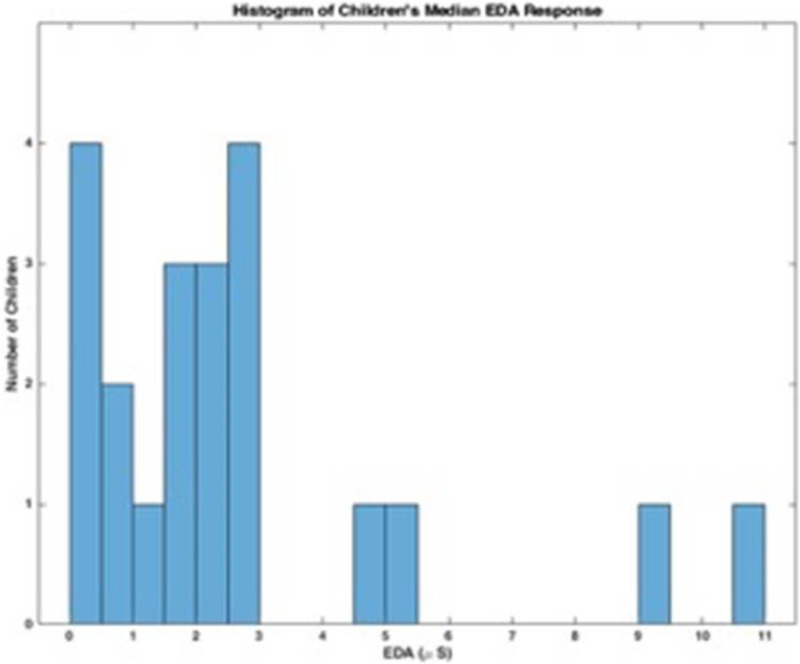
Distribution of EDA responses across participants.

**FIGURE 2:**
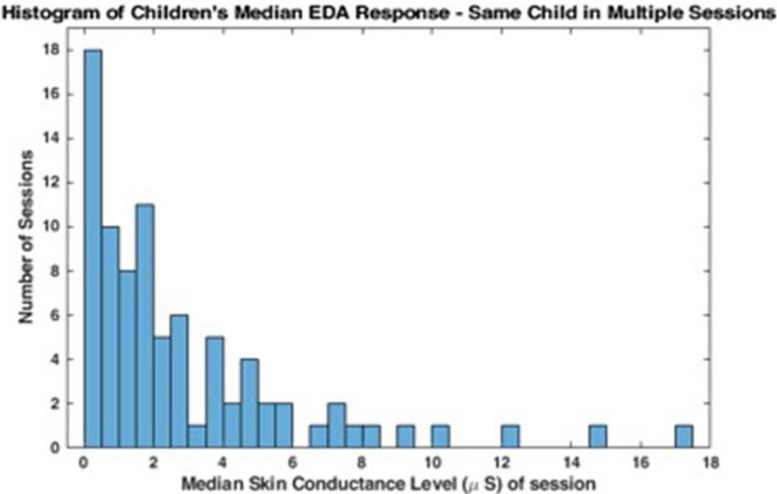
Distribution of EDA responses across sessions.

These data suggest that there may be a bimodal distribution in EDA; some children have lower baseline EDA responses and others have higher more variable responses. The histograms in Figures 1, 2 represent the median skin conductance level across sessions for all participants in this study. A non-linear filter was used to remove sensor drops (most likely from movement) before calculating the median value. The first 5 min of each therapy session was not evaluated to allow for sensor acclimation. Similarly, the last 2 min of data for each session was removed to avoid any zeroed data from early sensor removal.

### Insights

Data were examined using case study methodology, allowing for the development of insight that could impact future studies. Five insights were generated about the interaction of treatment and EDA from the 77 h of therapy.

#### Arousal Fluctuates Within a Treatment Session

**FIGURE 3:**
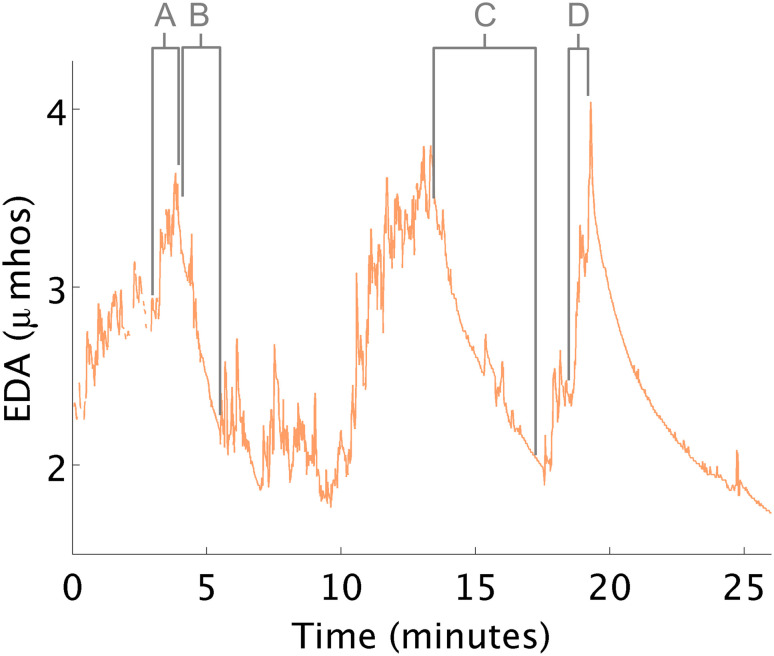
EDA changes during therapy. **(A)** EDA increases in response to a tunnel in the ball pit. **(B,C)** EDA decreases while in the ball pit. **(D)** EDA increases in anticipation of climbing onto a swing.

During this study, two challenges occurred creating anxiety (Figure 3). First, arousal increased upon seeing a tunnel in the ball-pit (see Figure 3A). The therapist explained that having the equipment out of its predictable environment can trigger this behavior. Second, the arousal increased right before the child climbed onto an elevated swing (see Figures 3B,C). When the child appeared distressed, there was a concomitant EDA increase. The therapist suggested that this increase was related to the child’s challenges with motor planning; anxiety was due to the motor response required to climb onto the swing. In fact, it is typical for EDA to increase when a person anticipates beginning a stressful or difficult task, even before they start the task.

Instances when therapeutic processes helped reduce arousal were also observed. For example, EDA decreased both times the child lay quietly in the ball-pit. The therapist hypothesized how these therapeutic events might affect the child, but the additional objective EDA measurement, made the therapist more confident about her interpretation of the child’s response.

#### EDA Increases When Engaging Large Body Muscles, Pulling Self Along Floor on a Scooter Board

**FIGURE 4:**
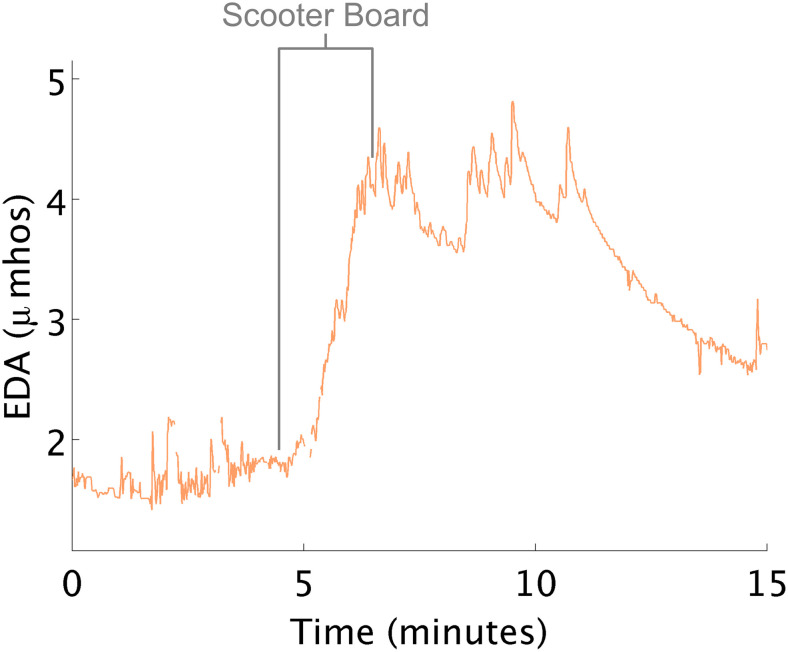
EDA increases while using the scooter board. EDA increases on scooter board.

At times, EDA changed in the opposite direction to that which the therapist expected. For example, therapists often attempt to decrease arousal using “heavy work”. Heavy work activities are those that maximally engage the proprioceptive system (e.g., large muscles and joint of the body) such as when children pull themselves along the floor on a scooter board. In this study, when children pulled themselves laying prone on the scooter board, increases in physiological arousal were consistently noted (Figure 4). Interpreting this response as high arousal is challenging, as hard physical work can also create these large increases in EDA (Hedman, 2014).

#### Child’s Arousal Decreases Unexpectedly

**FIGURE 5:**
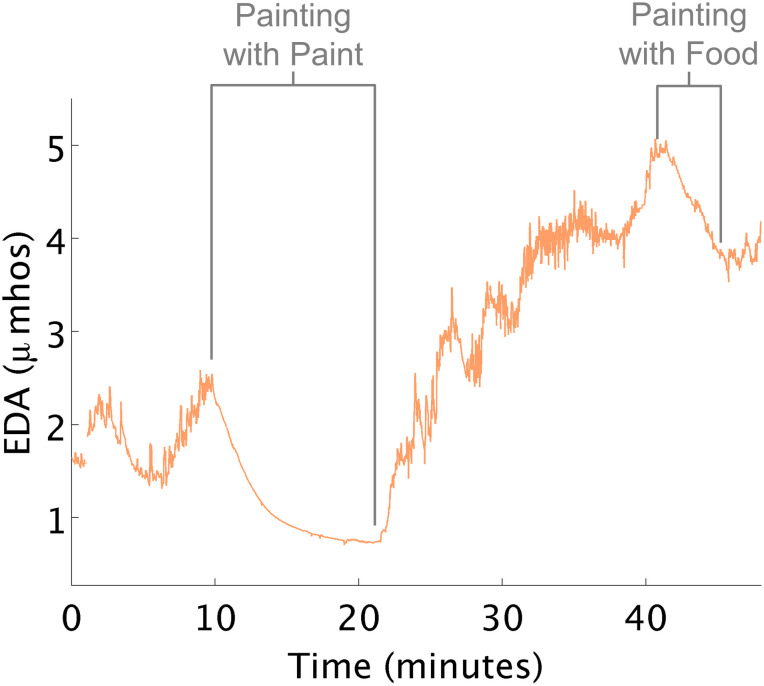
EDA decreases while painting. **(A)** EDA decreased while painting tile. **(B)** EDA decreased while painting with food.

Therapy sometimes reduced arousal unexpectedly. For example, in this instance, a child who was oversensitive to touch, taste, sound, and smell who consumed most of her food intake via a gastronomy-tube, would become overwhelmed when asked to eat or smell food. In the data below (Figure 5), the therapist first painted a tile with this child. Although this activity was not designed to affect her arousal, EDA decreased to a level lower than any other time in therapy. The therapist was surprised by this result and wondered if she could use this knowledge to help the child eat. Later, in therapy, they sat in a small room and painted with pudding on a sheet of paper. Like during the first painting episode, her EDA decreased, and she did not demonstrate overwhelmed behavior when food was present.

#### Using the iLs VoicePro Program Can Increase Arousal

While the above examples describe children who were overaroused, at times children demonstrated underarousal during occupational therapy. In these cases, children may seem tired and inattentive. They may struggle to pay attention to a task and may not be enthusiastic about the task at hand. When children’s arousal is too low, therapy activities are needed that raise arousal. There were several instances where children started therapy with low arousal marked by low EDA (often times in the early morning sessions).

**FIGURE 6:**
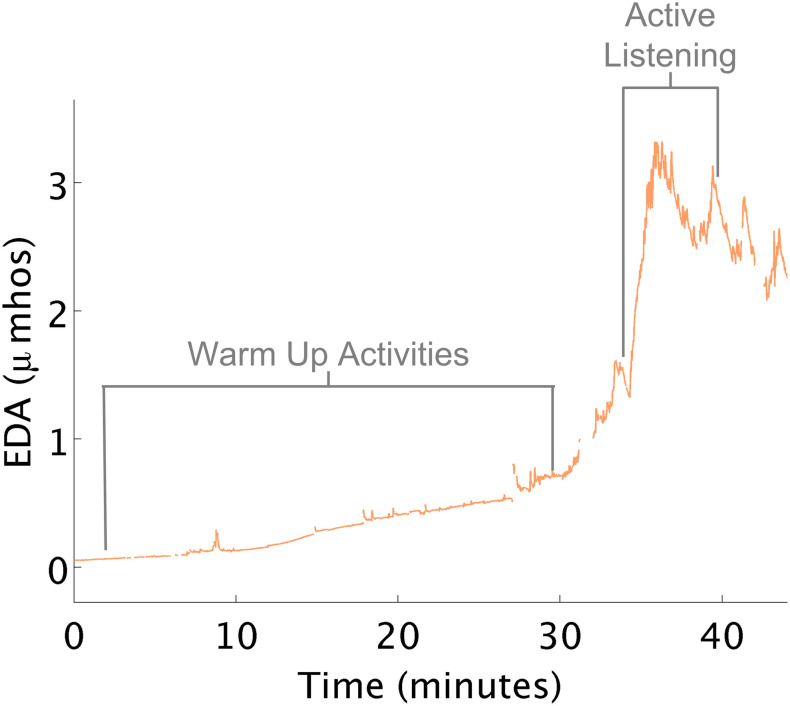
Increased EDA with voice pro program. **(A)** Physical activities that minimally increased EDA. **(B)** Voice Pro activity increased EDA.

Therapists attempted to increase the child’s arousal in preparation for learning and active participation in therapy. Playing in the ball-pit, swinging on a bolster swing, crawling through a tunnel, and jumping on a trampoline all appeared to have minimal to no effect on changing the child’s physiological arousal as expected (Figure 6). Near the end of the session, the therapist mentioned to the child that she would be using the iLs Voice Pro next, e.g., part of the Integrated Listening Therapy^®^ intervention used in occupational therapy. During this activity, the child wore earphones and heard her own voice as she talked. As she began this segment of her OT program, her EDA spiked. This event and its supporting data were shared with the therapist, who was surprised at the results that the iLs Voice Pro program helped increase the child’s arousal as marked by the increase in EDA. This data helped suggest a follow-up evidence based study (Schoen et al., 2015). For another child, the therapist scheduled the iLs Voice Pro program at the beginning of therapy rather than at the end of a treatment session to achieve increased arousal. In fact, during subsequent sessions, with the changed schedule, the child’s average EDA was maintained at a higher level than it had been in previous sessions.

#### Behavior Differs From Internal Arousal

**FIGURE 7:**
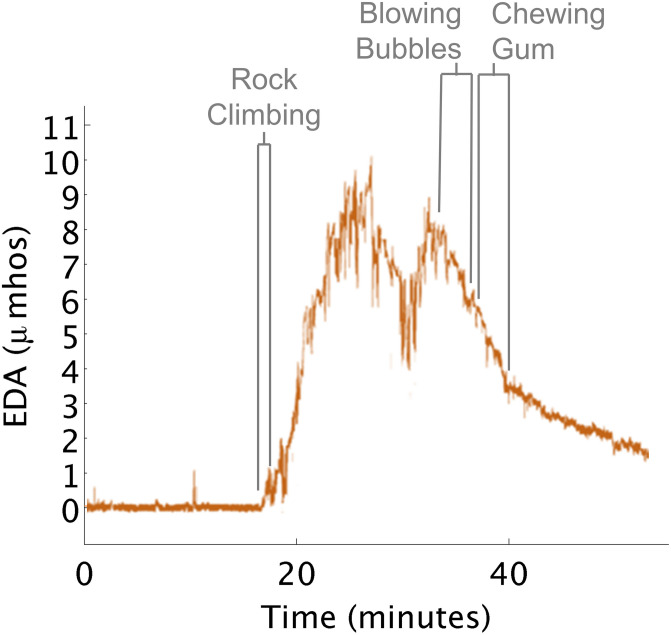
EDA increases during rock climbing. **(A)** Rock climbing increased EDA. **(B)** Blowing bubbles decreased EDA. **(C)** Chewing gum decreased EDA.

This insight highlights how outward behavioral responses can differ from a child’s internal state of arousal. Here, the therapist engaged a child in a rock climbing activity (heavy work) that she believed was going to decrease the child’s level of arousal (Figure 7). She continued with additional proprioceptive activities, (such as riding the zip line in a flexed position, and releasing grip to fall into the ball pit), under the assumption arousal needed to be further decreased. However, the child was actively complaining that he was tired and wanted to discontinue the session.

When the therapist saw the EDA data it suggested that the child’s physiological arousal was actually exceptionally high after rock climbing. The therapist hypothesized that the child wanted to disengage from the activities as a way of calming down. So the therapist went into the small kitchen where instead the child blew bubbles and chewed gum. During these activities, his EDA decreased close to the level seen before he engaged in rock climbing.

## Discussion

This study showed that ambulatory measurement of EDA with a wearable sensor was a feasible method for measuring physiological arousal in children with sensory processing challenges. Although concern is sometimes raised that measuring EDA *in situ* can alter an individual’s emotional experience (Lemos, 2008; Wrigley et al., 2010), that did not appear to be the case for this study. Ambulatory measurement of EDA was shown to be a viable method for interpreting arousal within an observational study of occupational therapy. We successfully measured 22 children’s arousal unobtrusively and *in situ* during 77 routine occupational therapy sessions. Children were able to engage in occupational therapy sessions and did not appear distracted by the sensor. No change in activities was required due to the sensors.

A contributing factor to the high success rate was the placement of the sensors on the bottom of the calf rather than the wrist or hand. In the pre-study pilot (*n* = 7), all children appeared bothered by the sensors on the hand and would often look at the sensors during therapy. Additionally, the children would move the wristband which interfered with data recording. This distraction was likely due to the sensor being in the child’s field of vision and on a sensitive part of the body. Sensors placed on the calf were not as noticeable and were out of the child’s immediate vision.

Recording data locally on the sensor and broadcasting data live is recommended in the future to prevent data collection from being obstructed by the child’s behavior such as crossing of the legs and being in a piece of therapy equipment that would block the view of the sensor to the computer (Hedman, 2011; Poh et al., 2012). This is now available on the current Empatica E4 device from the following website, https://www.empatica.com/research/e4/.

EDA data collected appeared to be meaningfully related to the activities in which the child was engaged. However, challenges exist in interpreting this physiological data, including issues with movement and determining specific internal processes that might affect a child at any given moment in time (Pugh et al., 1966; Cacioppo and Tassinary, 1990). With these caveats in mind, this study provides foundational support for future work using wireless EDA as a measure of children’s physiological arousal during therapy.

Results of this study suggest that physiological responses captured in the moment may be a more objective, accurate reflection of the individual’s arousal and response to intervention. The insights generated from this study show that by measuring ambulatory EDA, occupational therapists were able to redesign elements of their therapy. Therapists were able to understand how children became overaroused and what helped children calm them down. In several cases, therapists altered the therapeutic experiences of the children and thus affected the children’s state of arousal.

Traditionally, treatment research has focused on group averages and mean differences (Kravitz et al., 2004; Baldwin et al., 2008). This study suggests a method to explore and examine individual differences that can account for the variation in responses that individuals have (rather than grouping all data to create average scores). Some children have lower than average arousal; others have higher than average arousal when exposed to the same or similar situation (Schoen et al., 2008b). A ball-pit may help some children to calm down, while others may become overaroused. Each individual has his or her own responses to therapy activities, which may lead to unique emotional responses. Rather than attempting to erase or control for these differences, therapists can appreciate and take advantage of the variety of responses when they occur.

Arousal, however, is impacted by multiple factors. While the focus of this study was on the sensory-motor experiences of children in occupational therapy, the therapist’s feedback shed light on additional factors influencing the child’s arousal during the session, including emotional and cognitive features of the activities that may have impacted the child’s response. For example, the emotions associated with food for one child increased her arousal. When a cognitive component such as painting was added to the activity with food, her arousal was maintained at a lower level. Similarly the child whose arousal increased while engaged in the iLs Voice Pro task showed an increase in arousal due to the cognitive and social demands of this activity.

Thus, an increase in arousal does not fully explain a child’s experience. Whether a child is excited, anxious, or frustrated cannot be determined with EDA alone (Lang et al., 1998; Norman et al., 2016). A recurring question in physiological research is establishing a cause for increases or decreases in EDA. It is unclear as to whether physiological arousal increases because of a child’s body position or muscle activation (Pugh et al., 1966), the emotional challenge of transitioning to a new activity, an unknown factor or a combination of all three.

These findings have implications for other therapeutic applications of EDA. One application of EDA is its use as a biofeedback tool (Critchley et al., 2001). Research has shown performance in the workplace can be enhanced by real time feedback from a physiological sensor (Sano et al., 2015). Users have been found to be able to learn to recognize feeling states and associate such states with their physiology (van der Zwaag et al., 2013). Behavioral approaches that impact a child’s ability to self-regulate are common in occupational therapy practices for children with sensory processing challenges (e.g., Williams and Shellenberger, 1994; Kuypers, 2011). The goal of these strategies is to help children categorize subjective arousal states and use that knowledge to alter behavior. EDA offers an additional tool for recognizing changes in arousal that could be used to improve self-awareness and self-regulation.

## Conclusion

Thus, this research supports the literature showing that EDA is a reliable, interpretable, simple to use measure that has many applications and that has many options for recording sites (van Dooren and Janssen, 2012). This study showed that EDA data can be reliably and feasibly collected from the ankles. While applications vary, from EDA predicting self-reported emotional arousal (Fantato et al., 2013), stress recognition (Sano et al., 2015), or response to task difficulty (Fritz et al., 2014), this study was novel in that it was a significant first step in demonstrating the application and usefulness of physiological data from wearable sensors that might be used to inform occupational therapy treatment practice.

## Challenges

A variety of challenges are suggested by this research. The specific reason(s) for why EDA changed cannot be discerned with certainty, as the measurement of EDA alone does not explain everything about what is going on in a treatment session. Additionally, the valence of emotional state is not provided from the EDA data. Thus, inferences are made by comparing EDA data to segments of the treatment session on videotape, as well as interview of the therapist, but cannot be reliably explained by the EDA signal alone.

Another confound is that EDA can increase and decrease from causes other than sensory or psychological factors. Physical effort can also increase arousal. For example, EDA increased during a segment when the child moved across the ball-pit. This muscle activation is likely to increase arousal, but the child could also have been anxious or excited about the movement, which in turn would also increase EDA. Future research should attempt to measure and account for additional factors such as movement, temperature, speech, etc. that can increase EDA (Houtveen and de Geus, 2009). Questions of how much EDA increases from physical arousal versus cognitive or emotional arousal requires a device that detects both motion and temperature to partly separate these effects. Further, it is possible for a person’s EDA to change significantly with seizures, including non-convulsive seizures that may be not visible outwardly yet that may affect attention and activity. An individual who has “unexplained” large EDA peaks may have another undiagnosed neurological condition (Reinsberger et al., 2015).

## Future Work

This study illustrates how and when EDA can change during real-time occupational therapy intervention. Future work should compare EDA measured on the calf to other physiological measures such heart rate variability, and vagal tone. In addition, follow-up studies should evaluate the aggregated effects of physiological arousal from therapeutic activities. Understanding what specific therapeutic activities increase or decrease arousal within and across individuals would be desirable. Additionally, future work could focus on identifying the casual mechanisms within therapy. For example, what factors of the ball-pit are most helpful in reducing physiological arousal: body position, task, duration, etc. Overall, this article suggests a new lens to view occupational therapy sessions in real time, which can be used for further scientific investigation.

## Data Availability Statement

The datasets generated for this study are available on request to the corresponding author.

## Ethics Statement

The studies involving human participants were reviewed and approved by the Internal Review Boards of the Massachusetts Institute of Technology and Rocky Mountain University of Health Professions. Written informed consent to participate in this study was provided by the participants’ legal guardian/next of kin.

## Author Contributions

EH directed the research study from conceptualization, to data collection, data analysis, data interpretation, and manuscript preparation. SS and LM assisted in conceptualization, data collection, data interpretation, and manuscript preparation. RP assisted in conceptualization, data interpretation, and manuscript preparation. All authors contributed to the article and approved the submitted version.

## Conflict of Interest

EH was employed by mPath. RP developed the version of wearable sensors that were used in this study. She is the co-founder of Ematica, Inc., the company that now sells an updated version of this device. The remaining authors declare that the research was conducted in the absence of any commercial or financial relationships that could be construed as a potential conflict of interest.

## Footnotes

1 https://www.empatica.com/research/e4/
