# Supplementary material for: Wireless Measurement of Sympathetic Arousal During in vivo Occupational Therapy Sessions
Source: Front Integr Neurosci. 2020 Oct 29;14:539875. doi: 10.3389/fnint.2020.539875 (PMC7659428; doi:10.3389/fnint.2020.539875)
Supplement: Supplementary file 1 [file Data_Sheet_1.pdf]

Supplemental File: ASD Data from Dr Rosalind Picard's laboratory

Picard, R. (2020). EDA data from a sample of children on the autism spectrum. Unpublished manuscript. MIT Media Laboratory

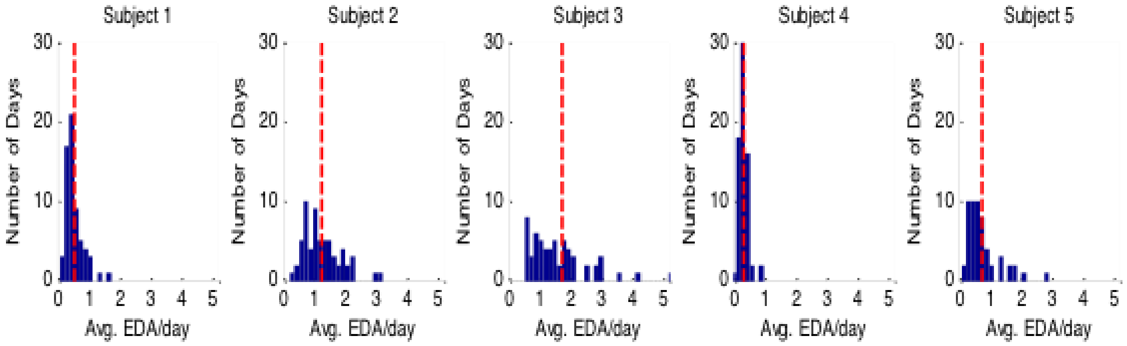

Dr. Picard produced these histograms from data collected on school aged children on the autism spectrum. These histograms show the mean EDA on the lower calf for five children on the autism spectrum, over 60 days, each child putting on the sensor when they went to school and taking it off before leaving school. The children were in the same school, so variations due to weather/activities/etc were comparable across the children for the 60 days of data.
